# A DPSIR Model for Ecological Security Assessment through Indicator Screening: A Case Study at Dianchi Lake in China

**DOI:** 10.1371/journal.pone.0131732

**Published:** 2015-06-24

**Authors:** Zhen Wang, Jingqing Zhou, Hugo Loaiciga, Huaicheng Guo, Song Hong

**Affiliations:** 1 School of Resource and Environmental Sciences, Wuhan University, Wuhan, Hubei, China; 2 Department of Geography, University of California Santa Barbara, Santa Barbara, California, United States of America; 3 College of Environmental Science and Engineering, Peking University, Beijing, China; Shandong University, CHINA

## Abstract

Given the important role of lake ecosystems in social and economic development, and the current severe environmental degradation in China, a systematic diagnosis of the ecological security of lakes is essential for sustainable development. A Driving-force, Pressure, Status, Impact, and Risk (DPSIR) model, combined with data screening for lake ecological security assessment was developed to overcome the disadvantages of data selection in existing assessment methods. Correlation and principal component analysis were used to select independent and representative data. The DPSIR model was then applied to evaluate the ecological security of Dianchi Lake in China during 1988-2007 using an ecological security index. The results revealed a V-shaped trend. The application of the DPSIR model with data screening provided useful information regarding the status of the lake’s ecosystem, while ensuring information efficiency and eliminating multicollinearity. The modeling approach described here is practical and operationally efficient, and provides an attractive alternative approach to assess the ecological security of lakes.

## Introduction

Many lakes and reservoirs in China have suffered severe eutrophication in recent decades. A national monitoring database revealed that eutrophication occurred in 41% of all major lakes in the 1980s, and 77% in the 1990s [1]. A national survey that started in 2006 indicated that 85% of the lakes with a surface area larger than 10 km^2^ were eutrophicated at different levels of severity [2]. Eutrophication causes the degradation of aquatic ecosystems, with frequent algal blooms, which endanger water supplies and fisheries in the impacted lakes. The public and scientists have combined to appeal for a safer and healthier water-quality environment in lakes throughout China. This prompted the Ministry of Environmental Protection of China (MEP) to launch a nationwide project in 2008, the Ecological Security Assessment for Lakes and Reservoirs (ESAL). ESAL uses “ecological security” as the core concept to address the relationships between lakes/reservoirs and social and economic sectors as ecosystems. ESAL’s purpose was to obtain a better understanding of the past and current status of lakes and the stresses imposed on them by human activities. Dianchi Lake was one of the pilot lakes selected by ESAL to establish an analytical framework that could be expanded to other key lake assessments.

The goal of ecological security is to conserve ecosystems in an unthreatened state while still experiencing human activity, and to provide the necessary resources to maintain their health and ability to adapt to environmental changes [3–7]. Current ecological assessments of lakes and reservoirs rely primarily on an index-based assessment approach [4,8,9] or the ecological footprint approach [10]. In most cases, the indicators selected for index evaluation of lake and reservoir ecosystems are critical to the success of conservation programs. The judicious selection of indicators increases the scientific credibility of ecological assessments [11]. In current practice, indicators for ecological assessments are often selected based either on historical practice or on the judgment of experts [12], which may bias the assessment results due to data multicollinearity (i.e., statistical redundancy) or the duplicitous use of information. We propose an integration of the ecological security assessment with a statistical indicator selection procedure that measures the inter-relation of ecological indicators as part of the indicator selection process.

The first part of this paper presents a brief introduction of the ESAL assessment framework for Dianchi Lake, followed by a description of the indicator selection process. The second part demonstrates the merits of the proposed model for lake ecosystem assessment using a case study of its implementation in the Dianchi Lake watershed.

## Ecological security assessment model

### 2.1 DPSIR framework for ecological security

**Fig 1:**
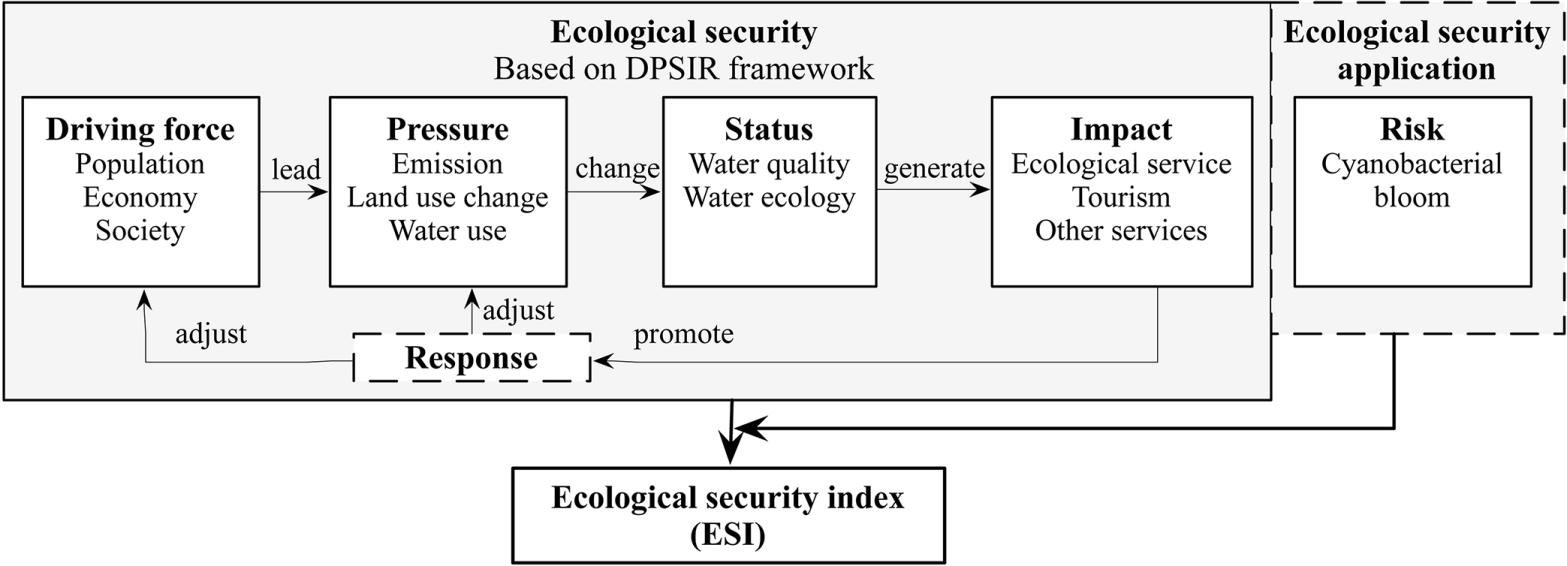
Conceptual model and evaluation method of ecological security assessment.

The concept of ecological security emphasizes the sustainability of ecosystems, focusing on reducing the probability or risk of ecological disaster caused by human-induced stresses [13]. In the specific case of Dianchi Lake, it is essential to investigate the lake itself and the surrounding social and economic sectors, which are often the driving forces behind ecological degradation [12]. As seen in Fig 1, a change in a lake’s ecological health, driven by anthropogenic activities, generates benefits or losses of ecological services. One task of ESAL was to identify the dynamic links that exist between a lake ecosystem and its surrounding social and economic sectors. In this study we introduced disaster evaluation as part of the ecological security assessment of lakes. The DPSIR indicators are as follows:

*Driving-force* (D) indicators describe the social and economic developments in the communities surrounding the lake as well as the corresponding changes in lifestyle, consumption, and production patterns. These indicators include the regional population, population density, gross domestic production, per capita income, Gini coefficient, and other factors that directly or indirectly influence lake ecosystems and water quality.

*Pressure* (P) indicators measure the impacts that human activities exert on a lake, especially on its water quality and quantity. With regard to water quality, pollutant emissions are the main stressor. Thus, various sources of pollutants should be considered. Conflicts between human and aquatic functions often occur in water-deficient areas [14]. Therefore, the total water-resource utilization, water-quantity ratio of inputs and outputs in a lake, per capita water-resource use, and ecological water demand and related data should be included in any index of a lake’s ecological evaluation.

*Status* (S) indicators reflect the ecological health and include water quality and water ecological factors. Water quality indicators can be obtained from conventional water monitoring. The water-ecology indicators reveal both the community and system status. The phytoplankton biomass (B_p_), zooplankton biomass (B_z_), large zooplankton biomass (B_macroz_), small zooplankton biomass (B_microz_), ratio of zooplankton and phytoplankton biomass (B_z_/B_p_), and ratio of large zooplankton and small zooplankton biomass (B_macroz_/B_microz_) are community ecological indicators. Energy quality (E_x_) and energy quality structure (E_xst_) are used to evaluate a system’s ecological status [8,15].

*Impact* (I) indicators represent the variation in lake services (e.g., fishery output and tourism) caused by a lake’s change of status.

*Risk* (R) indicators are used to express the chance of the occurrence of an ecological disaster. In eutrophic lakes, the major risk is an algal outbreak, with consequent health hazards to humans and ecological hazards to organisms. The indicators are bloom duration, time, area, and the population or biomass affected through drinking water or exposure to the bloom.

In Dianchi Lake, nutrient concentrations have largely surpassed the commonly accepted threshold that triggers a cyanobacterial bloom [16,17], and weather conditions are the key factor that influences the occurrence of a bloom [18]. Formula (1) can be used to quantify the risk of a cyanobacterial bloom:

$$x_{i,r}=1-\frac{n_{i}-n_{Ti}}{n_{i}}\times \frac{n_{i}-n_{TPi}}{n_{i}}\times \frac{n_{i}-n_{TNi}}{n_{i}}$$ (1)

where *x*_*i*,*r*_ is the risk of a cyanobacterial bloom in the *i* ^th^ year, which is used as the indicator variable for the risk component (R) in the DPSIR model; *n*_*i*_ is the number of all data entries in the *i* ^th^ year; *n*_*Ti*_ is the number of data entries in the *i* ^th^ year with temperature below the lowest temperature recorded when cyanobacterial blooms occur; and *n*_*Pi*_ and *n*_*Ni*_ denote the number of data entries in the *i* ^th^ year with total phosphorous (TP) and total nitrogen (TN) concentrations, respectively, below the lowest concentration observed when cyanobacterial blooms occur. The risk *x*_*i*,*r*_ in Eq (1) varies between 0 and 1.

### 2.2 Data screening

For a large assessment program, the DPSIR model data are chosen from a number of existing databases, which may contain large amounts of information, with a variable degree of usefulness. The indicators used in an ecological security assessment should meet some basic criteria if they are to be considered in the DPSIR method. These are:

◆ 1. Relevancy: indicators should reflect changes in ecological quality, status, management, or other security domains.
◆ 2. Availability: the indicator data should be available, accessible, and consistent within the period of analysis.
◆ 3. Independence: indicators must be independent of each other to eliminate multicollinearity (statistical redundancy or duplicity).
◆ 4. Representativeness: each indicator used in the model must represent a category or phenomenon of its own, and must provide superior information to other indicators in a similar category.

Criteria 1 and 2 can be easily achieved by searching every available database for their period of applicability and their information content. A correlation test can be used to evaluate the independence of data sets. If a significant correlation is identified among indicators, multicollinearity may arise in the ecological assessment model, which would affect the accuracy of the ecological security index. Data multicollinearity is common among socio-economic indicators. When such statistical redundancy occurs, one or a few indicators with the highest information content should be selected from among the correlated indicators. A principal component analysis (PCA) is helpful for selecting the most significant ecological indicators. The field studies were carried out in the monitoring stations controlled or supervised by Kunming Environmental Monitoring Center. Kunming Environmental Monitoring Center has all authority to permit our access to the data of Dianchi Lake.Detailed data screening process as well as an example was given in S1 Text.

### 2.3 Ecological security index

The ecological security index (ESI) is herein proposed to evaluate a lake’s deviation from a background condition (the monitoring data for 1988 was chosen as the background condition in this work). To calculate the ESI, the data are first standardized using Eqs 2 and 3 to transform the DPSIR data into “the-higher-the-better” numbers by normalization as follows:

$$x_{i,j}^{'}=\frac{x_{i,j}}{x_{1988,j}}$$ (2)

when the indicator *x*_*ij*_ has a value reflecting improved conditions relative to the 1998 baseline value (*x*_*1998*,*j*_); and:

$$x_{i,j}^{'}=\frac{x_{1988,j}}{x_{i,j}}$$ (3)

when the indicator *x*_*ij*_ has a value reflecting a worsening condition relative to the 1998 baseline value. In Eqs (2) and (3), *i* denotes the year and *j* denotes the ecological indicators. The normalized variables *x′*_*ij*_ defined by Eqs (2) and (3) lead to DPSIR index values and an ecological security index (ESI), in which a rising (declining) magnitude reveal an improving (worsening) condition, thus “the-higher-the-better” numbers appellative are used to characterize them.

The index of each DPSIR component is calculated with the following averaging formula:

$$Index_{k,i}=\prod_{j\in k}{\left(x_{i,j}^{'}\right)}^{w_{k,j}}$$ (4)

where, *k* = 1, 2, 3, 4, 5, which denotes the component of the DPSIR model, namely, driving-force, pressure, status, impact, and risk, respectively. *w*_*k*,*j*_ is the weighting for the *j*-th indicator of the *k*-th component. For example, the weighting for a component *k* with 3 indicators is (1/3, 1/3, 1/3) and $Index_{k,i}=\prod_{j\in k}{\left(x_{i,j}^{'}\right)}^{1/3}$.

The ESI for the *i*-th year is calculated using the following averaging formula:

$$ESI_{i}=\prod_{k}{\left(Index_{k,i}\right)}^{w_{k}}$$ (5)

where *ESI*_*i*_ represents the ecological security index of *i*-th year. *w*_*k*_ is the weighting for the five DPSIR components, that is (*w*_*k*_ = 1/5, 1/5, 1/5, 1/5, 1/5, for *k* = 1, 2, 3, 4, 5, respectively).

## Case study: application to an assessment of Dianchi Lake

### 3.1 Background of Dianchi Lake

**Fig 2:**
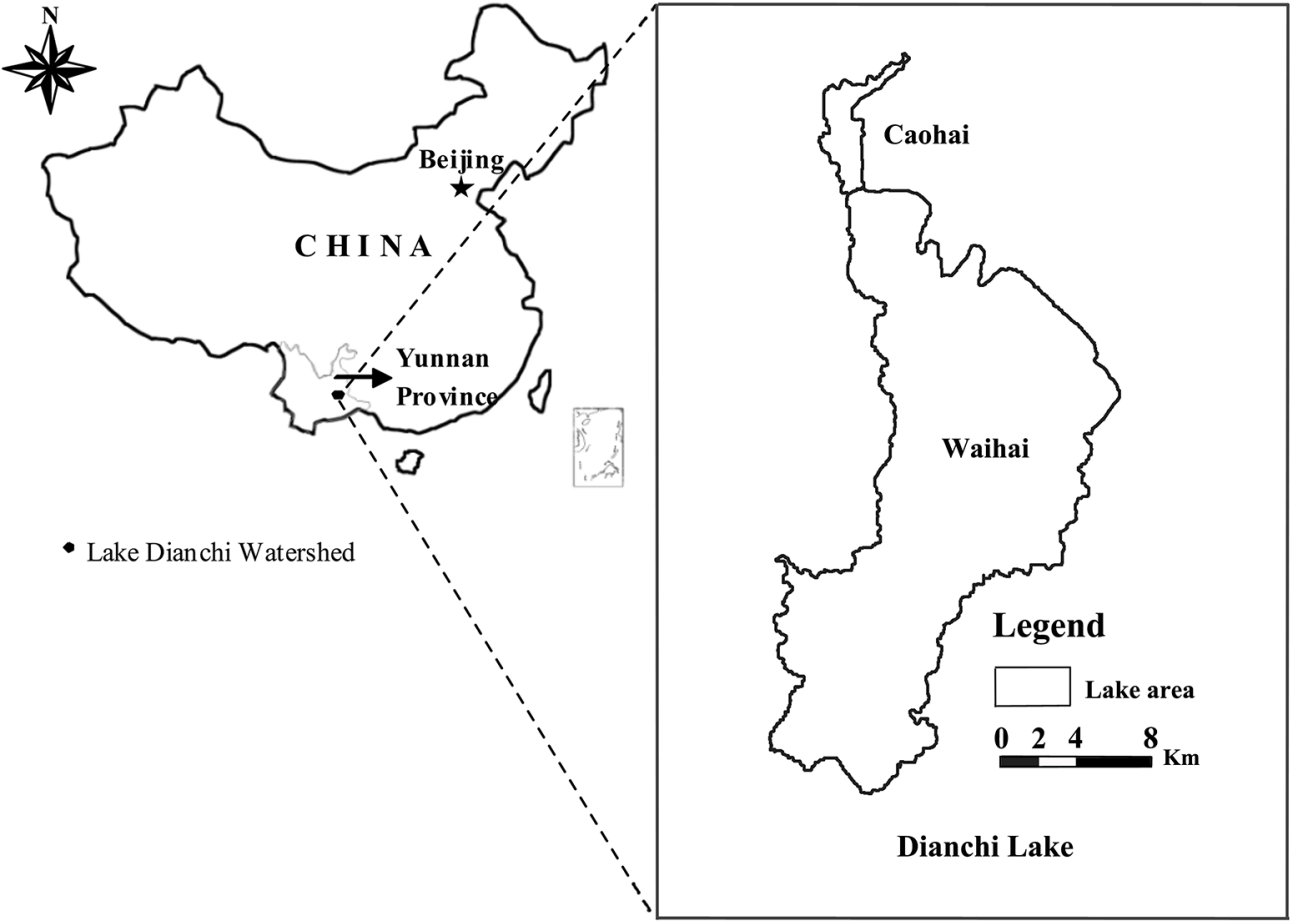
Location of Dianchi Lake.

Dianchi Lake is located within the City of Kunming, the capital of Yunnan province, at an altitude of 1887.4 m (Fig 2). It is the sixth largest lake in China, with a water surface area of 292 km^2^, extending from 24º28′ to 25º28′N and 102º30′to 103º00′E. Rapid urbanization and industrialization in Kunming since the 1980s have resulted in increased pollution discharges into Dianchi Lake. As a result, both the water quality and ecosystem health have deteriorated dramatically. The area covered with aquatic plants was approximately 20% of Dianchi Lake surface in the 1970s [19]. By the late 1990s only 1.8% of the water area was covered by aquatic plants [20]. Over the last 30 years, 29 species of aquatic plants and 21 species of indigenous fishes went extinct in the lake. Meanwhile, cyanobacteria, specifically *Microcystis aeruginosa*, became the dominant algae, and has bloomed throughout the year after 1998 [18]. Dianchi Lake gradually lost its ecological service functions for drinking water supply, fisheries, and irrigation. The ESAL project’s objective for Dianchi Lake was to evaluate its current status and aid decision making in the “12^th^ five year national environmental plan” as well as in the “Dianchi Watershed Planning Project”.

### 3.2 Data screening in the Dianchi Lake watershed

**Fig 3:**
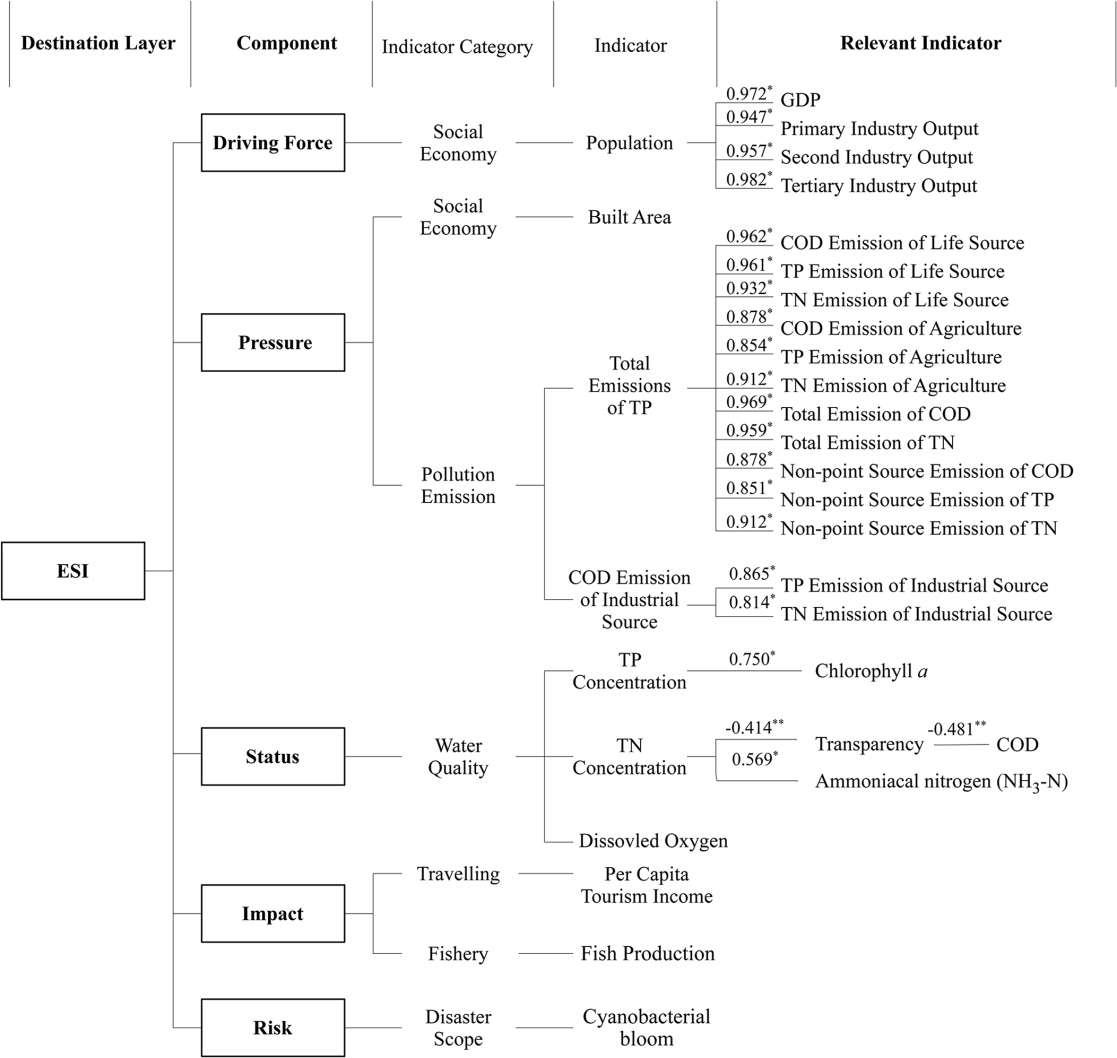
Data screening of Dianchi Lake ecological security assessment. (* Correlation is significant at 0.01 level (P = 0.01); ** Correlation is significant at 0.05 level (P = 0.05)).

Data were obtained from the local bureau of statistics, environmental monitoring stations, and fisheries authorities. Data that did not cover the period from 1988 to 2007 were excluded. The data screening process is shown in Fig 3. The numbers in Fig 3 denote the correlation between indicators and relevant indicators.

Driving (D) factors, such as population growth and economic development, were strongly correlated in the past 20 years. The PCA indicated that one indicator provided enough information as Fig 3 showed. Population growth explained the rapid urbanization and industrialization in the Dianchi Lake watershed [21], whereas economic activity did not explain the population increase. Thus, population was selected as the sole driving factor.

The area of construction land, emission of TP, and chemical oxygen demand (COD) from industrial sources were selected as the pressure (P) indicators. The area of construction land has expanded substantially to fulfill the residential and industrial building needs. The emission of TP was due to industry and agriculture. More recently, industrially derived TP has been reduced to become a minor source of pollution. Agricultural sources of TP are more difficult to control, and have become the most harmful regular pollutant of aquatic ecosystems in the study area. Most COD from industrial sectors was emitted into the Caohai area, causing foul odors and the mass death of fish. These three indicators (construction area, TP, and COD) constitute three pressure pathways from the increasing population on the lake’s ecosystem.

Status (S) indicators include TP, TN, and Dissolved Oxygen (DO) concentrations. The TP concentration is possibly the limiting factor of phytoplankton growth (correlation coefficient of the TP concentration to Chlorophyll *a* (Chl-*a*) = 0.75). The TN concentration was used as the status indicator instead of COD because it had a more important role in phytoplankton growth. DO is vital to aquatic organisms. When cyanobacterial blooms occur, DO may drop dramatically, leading to the suffocation of aerobic aquatic species, especially fishes.

Fish production and per capita tourism income were chosen as the impact (I) indicators, without screening, due to the lack of consistent data for evaluation. Ecological degradation, especially cyanobacterial blooms, leads to a reduction in fishery production. Foul odor, death of fishes, and other problems caused by pollution also reduce the economic output from tourism at Dianchi Lake. The cyanobacteria bloom index (formula 1) was used as the risk indicator.

### 3.3 The ESI index

**Fig 4:**
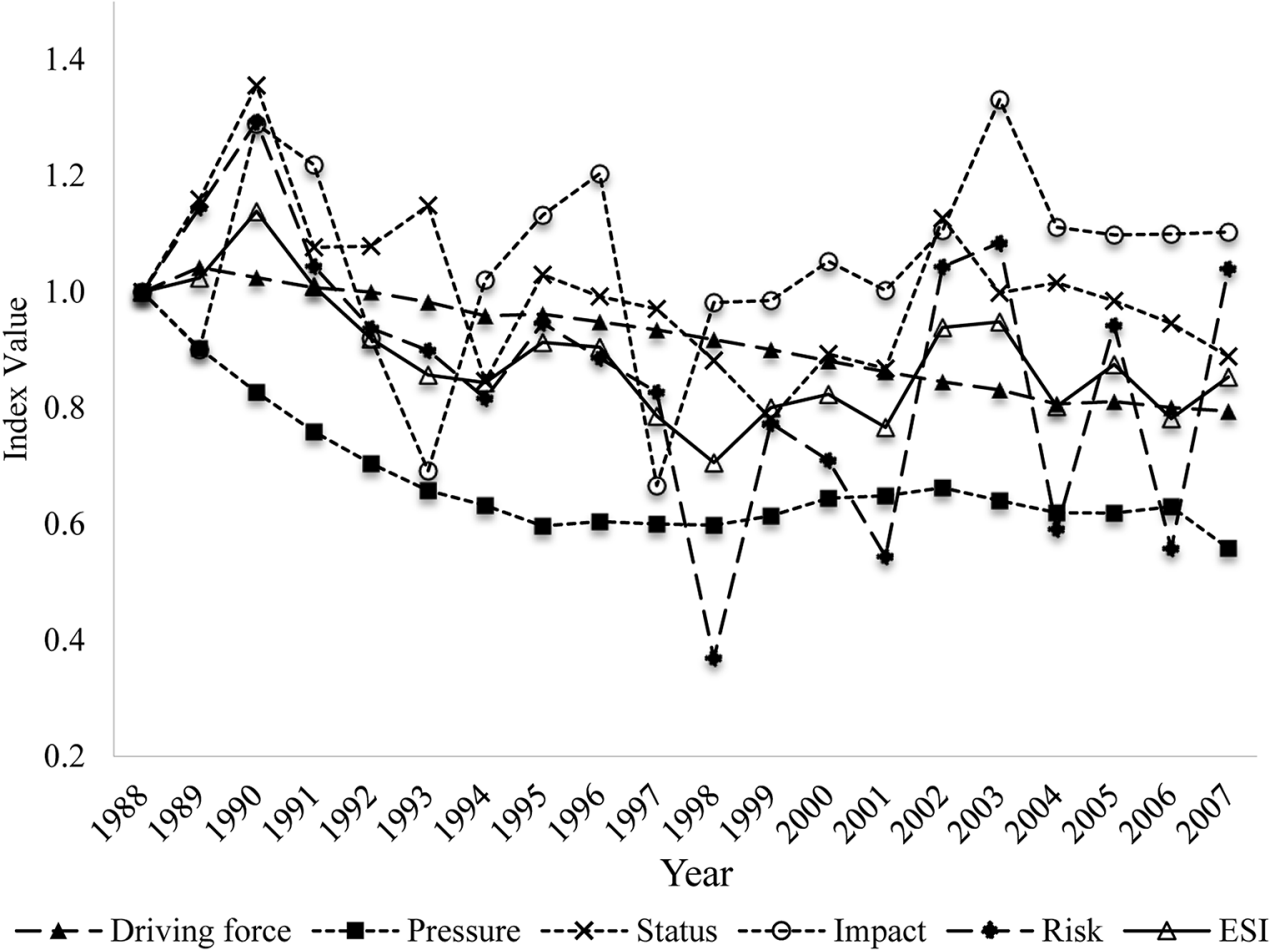
Ecological security index and DPSIR indices of Dianchi Lake.

**Fig 5:**
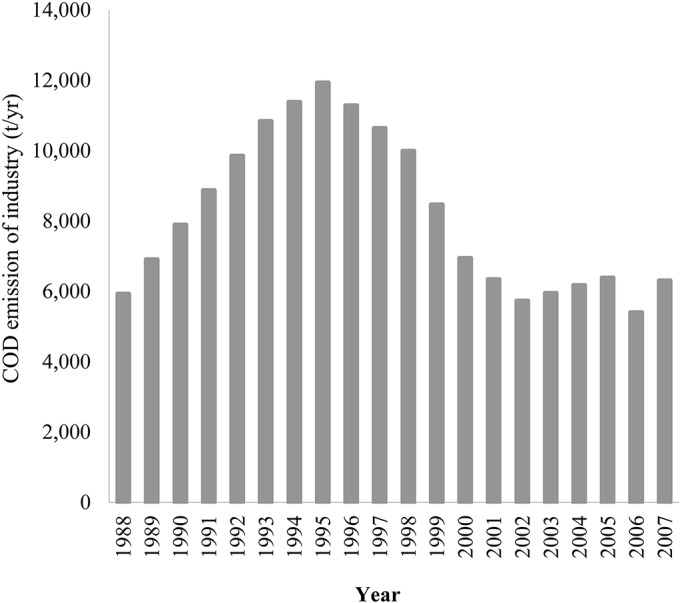
COD emission by industry during 1988–2007.

**Fig 6:**
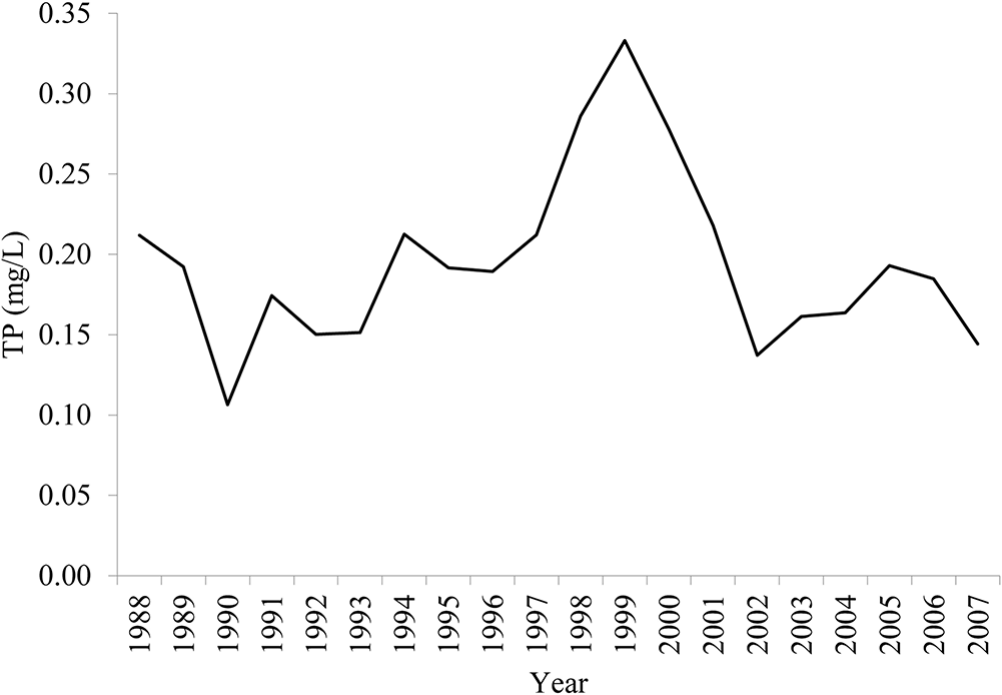
TP concentration in Dianchi Lake.

**Fig 7:**
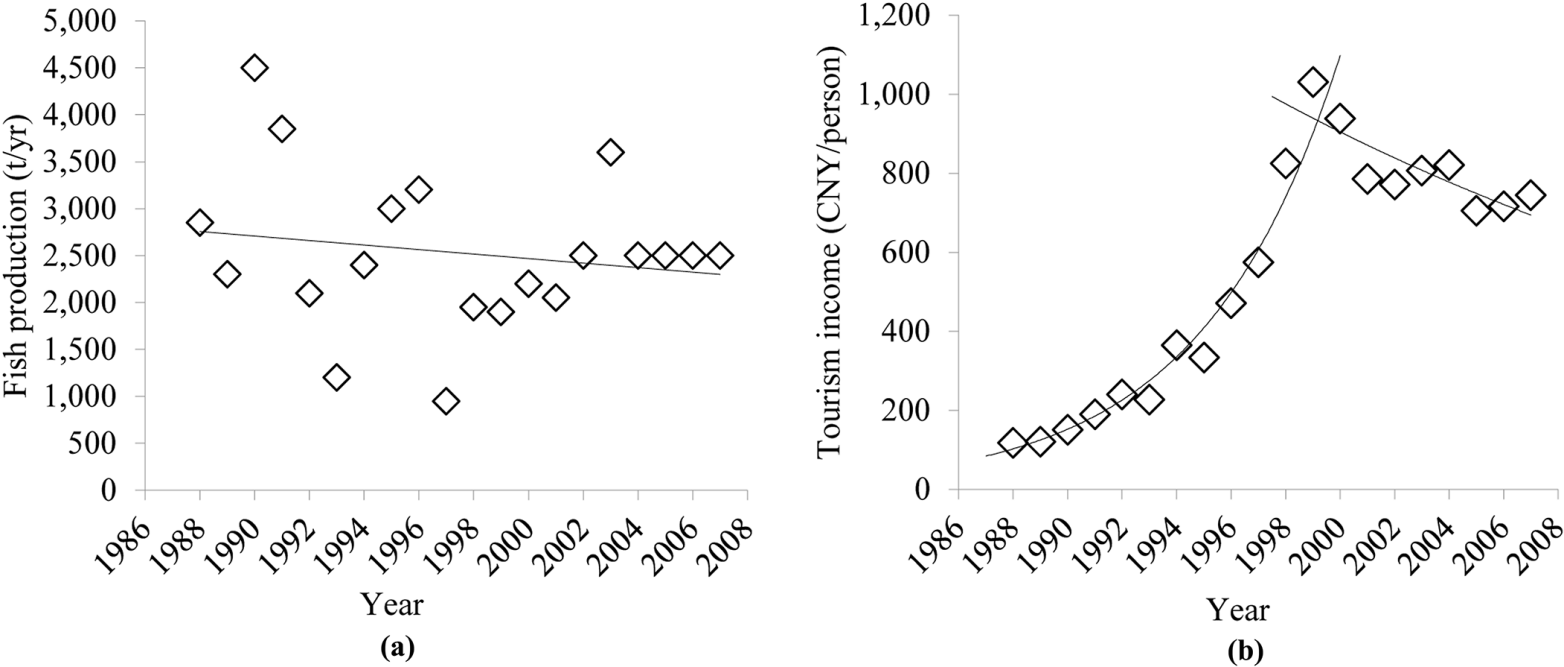
Fish production and per capita tourism income in the Dianchi Lake watershed.

The calculated ESI and the indices of the components of the DPSIR model are shown in Fig 4. The ESI indicates that Dianchi Lake exhibited a fluctuant V-shaped ecological security trend. In the period 1988–1998, the ESI declined and reached the lowest value (0.7061) in 1998. In the following decade, the ESI increased slightly, exhibiting erratic fluctuations. The driving-force indicator declined steadily over the period of analysis (1988–2007) and reached its lowest value (0.7947) in 2007. The pressure indicator had low values in the period around 1995–1998 and in 2007, as shown in Fig 4. Industrial COD emissions, nutrient loading to Dianchi Lake and the area of construction land together shaped the pressure indicator. The local government started to cut down on industrial COD emissions in preparation for the International Garden Festival (IGF) in 1999. Industrial COD emissions were identified as the main reason for the foul odor and scenic degradation in Caohai at that time. Four wastewater treatment plants were built and came into operation in 1995, 1997, 2002, and 2005. Therefore, industrial COD emission started to decline in 1996, as Fig 5 shows. In contrast, the sources of agricultural pollution did not receive sufficient attention. The flower-planting area in Kunming expanded rapidly after 1995 due to the rising market prices of flowers. The flower-planted area increased from 567 hm^2^ in 1996 to 6900 hm^2^ in 2007. Simultaneously, the application of fertilizer used for flower growing reached 567 kg·hm^-2^·yr^-1^, i.e., three times the level used in wheat fields. As a result, the nutrient loading to Dianchi Lake increased. The area of construction land also increased to meet residential, commercial, and industrial needs. Combining the favorable changes in COD emissions with the worsening nutrient loading and changes in land cover, the pressure index experienced a slight favorable turn around 1995–1998, but declined slightly during 2002–2007. The status, impact, and risk indexes displayed similar patterns to the ESI, with the lowest indicator values in 1997–1999. There are several reasons for this. (1) The TP concentration increased dramatically from 1997 to 1999 (see Fig 6). This was identified as the main reason for cyanobacterial blooms at that time. Emerging environmental protection measures, including a new industrial wastewater treatment plant and dredging in Caohai, were implemented before the IGF opened in 1999. Other long term polices for environmental protection were initiated at about the same time. As a result, the TP concentration began to fall after 1999. (2) There were two impact indicators: fish production reached its lowest outcome at 1997, and per capita tourist income reached its point of inflexion in 1999 (see Fig 7). Noticeably, per capita tourist income started to decline after 1999 in Dianchi Lake’s scenic area due to the impact of the frequent cyanobacterial blooms on recreational activities. (3) The risk index reached its worst level in 1998 (see Fig 4: a lower risk index value indicates a worsening condition, as implied by Eqs (2) and (3)). Compared to the seasonal events during April to November in previous years, 1998 was the first year when cyanobacterial blooms occurred year round. The variability of the DPSIR components increased over time, which reflects the influence of human activities on ecological security through complex biochemical cycles.

### 3.4 Discussion

**Fig 8:**
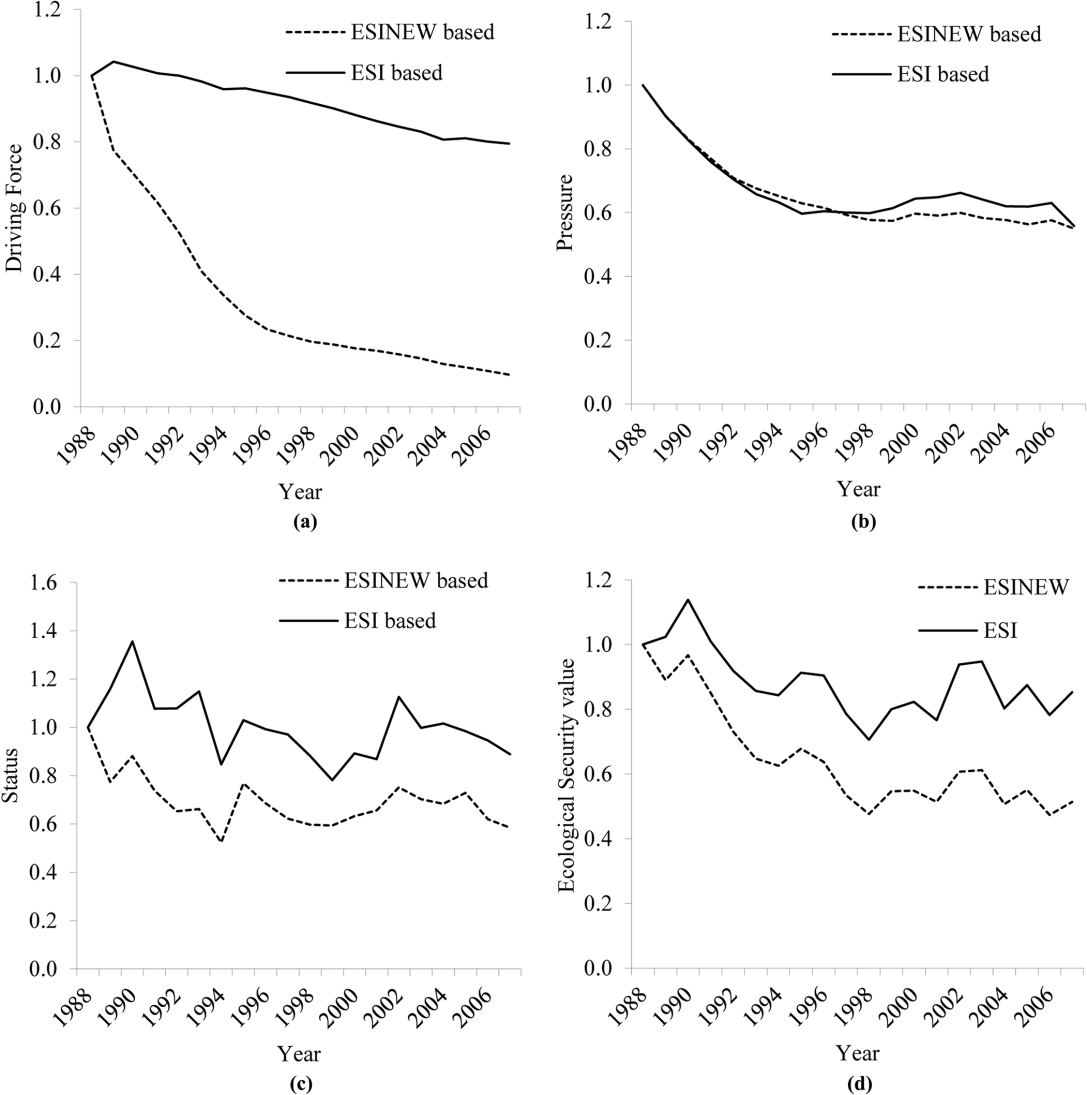
Evaluation of Driving force (a), Pressure (b), Status (c), Ecological security index (d) with and without data screening. (Notice that a lower value of an index implies a higher risk).

Data screening is essential to exclude redundant information and to determine the accuracy of ecological assessments. To demonstrate this assertion, the ESI was calculated without data screening (herein called the ESINEW), and was obviously lower than the previous results, as depicted in Fig 8. The key reason why the ESI performed better than the ESINEW was the multicollinearity of the driving force factors in the latter index. The strong correlations among population and economic indicators (correlation coefficient > 0.95, p < 0.05) provided redundant information, which undesirably enhanced the decreasing trend into steeper curves for the indicator shown in Fig 8. Data screening was useful to produce “unbiased” information with less data, i.e., similar trends in the driving force (Fig 8A), pressure (Fig 8B), status (Fig 8C) and ecological security (Fig 8D) indexes had R^2^ = 0.79, 0.90, 0.71, and 0.86, respectively (linear regression coefficient of determination, excluding 1988.). In brief, data screening in an ESI will eliminate the interference of excessive information, and assist in the diagnosis of various problems. It is clear that rapid urbanization caused a population increase in the watershed area, as well as the need for land and economic activity, and this was the motive power for deterioration of the lake environment. Therefore, in addition to routine end-of-pipe treatments and lake water quality management, long-term strategic population control/evacuation from the upstream of Dianchi Lake, as opposed to the current plans for a large population expansion, will be important [21]. In addition, land use control and policies to promote an adjustment of industrial structure should also be implemented alongside the existing lake management policies.

To eliminate just the redundant information, using the principal components as indicators might be a better alternative. It can transform the original data into uncorrelated components and use them to calculate the ESI, but it has a disadvantage in that sometimes it is difficult to explain the realistic meaning of the components. Compared our work with others’ findings in Diachi watershed, we had a longer monitor (1988–2007) than the literature [22] (1999–2007). And very similar trends were found during 1999–2007, although different indicators between our work and [22] were integrated into the DPSIR framework. Due to lack of research on ecological security in this area, we compared the results with an ecological health index study from [23], because the ecological health and ecological security is somewhat correlated. [23] had different evaluation technology and used different indicators from 1998–2001. But same decease trend of the indexes were found. These comparisons indicated that our model results were as accurate as other studies. Other ecological security or ecological health index focused on the spatial difference of Dianchi Lake, lacking long time series [24]. Compared to the literature regarding data screening [8, 11], our data screening process was an executable method to select indicators for assessing socio-economic threats and the current status of the lake. Most indicators used in this model are easy to obtain, which extends the model’s range of applications.

This method also has some limitations. First, it still relies on a subjective judgment for selecting relevant indicators from a database and categorizing them to suit the DPSIR framework. A combination of a group decision and Niemeijer and de Groot’s causal network framework [11] is likely to be a good solution to reduce the subjective bias. Second, a large sample size (preferably >20) is essential for conducting the statistical analysis. Therefore, long term continuous monitoring (>20 years or seasons) is needed. It is difficult to find such data in China, even for some key lakes/watersheds, which limits the model’s application. Due to the lack of monitoring, most data used in this study were aggregated. If applicable, extending our method with a spatial approach to assess the spatial distribution and variety would be more useful for pinpoint management.

## Conclusions

The ecological security of a lake requires an understanding of the integral health of the lake’s ecosystems, allowing the lake to consistently provide beneficial services and avoid ecological disasters caused by anthropogenic activities. Ecological security can be interpreted as a broadly defined sustainability, consisting of dynamic and beneficial cycles of human-environment interactions. To assess the variability of a lake’s ecological security requires large monitoring networks that track social, economic, and environmental-ecological components. Useful information can then be selected and utilized to derive unbiased ecological security indexes.

Eleven of thirty-two indicators were selected in the Dianchi watershed to assess the lakes ecological security status. Dianchi Lake experienced deterioration in 1988–1998 and a fluctuating recovery in 1999–2007. Efforts to control emissions and water quality have mitigated the deterioration of Dianchi Lake, to which human activities had predominantly contributed. Cyanobacterial blooms remain a high risk and continue to threaten Dianchi Lake. The application of the DPSIR model, combined with data screening, showed that it could eliminate the nefarious effect of data multicollinearity and provide useful information to assess the ecological security status of a lake. This work has produced a preliminary ecological security assessment for Dianchi Lake, China. Several associated studies are in progress: (1) the continuous monitoring of aquatic biological indicators; (2) the early warning of cyanobacterial blooms by building a more accurate risk evaluation model, considering weather conditions and aquatic biological indicators; and (3) an assessment of the ecological services affected by ecological degradation.

## Supporting Information

S1 Text Data screening process and example. (DOCX)
