# Supplementary material for: A DPSIR Model for Ecological Security Assessment through Indicator Screening: A Case Study at Dianchi Lake in China
Source: PLoS One. 2015 Jun 24;10(6):e0131732. doi: 10.1371/journal.pone.0131732 (PMC4479557; doi:10.1371/journal.pone.0131732)
Supplement: S1 Text — (DOCX) [file pone.0131732.s001.docx]

**S1 Text. Data screening process and example**

**1. Data screening process**

*Step 1, list all indicators that may be relevant with the ecological security (relevancy)*

Here, we used brainstorming method to enumerate all possible indicators. Most indicators are observed or reported in literatures. These indicators were mainly listed in 2.1. But at this step we did not know whether these data were available or not, and whether the available data would meet the requirement of the assessment or not.

*Step 2. Filter out indicators without enough data (availability)*

Here, we use only one criterion to filter out the indicators after the data collection. That is, the data of an indicator must have continuous monitor and cover the year from 1998 to 2007. Due to lack of data sharing mechanism, we could not collect all data we wanted. This is the main reason we have to consider the availability.

*Step 3. Use the correlation analysis to identify how much these data correlated, thus support the data screening (Independence)*

Pearson correlation analysis is a common model for identifying correlation between indicators. Pearson correlation coefficient *R* is calculated as the formula (1).

(1)

The absolute value of R indicates the magnitude of correlation. If R is equal to 0, the indicators are absolutely uncorrelated with each other. Namely, they are independent. If R is equal to 1, the indicators are completely correlated namely dependent.

If indicators have significant correlation, there is possible multillinearity that will affect the accuracy of the assessment. We could classify the indicators into several groups, the indicator in which has high R-value. Namely, we separated the indicators into several independent groups.

Step 4. Choose an indicator as a representative of a group (representativeness)

Basically, we use the principal component analysis to choose a representative indicator, which has high correlation with the principal components. First, we standardize the data by removing the indicator’s mean value to form a new zero mean dataset. Then, we calculate the covariance of the new dataset as formula (2)

$C=\left[\begin{matrix} \sum X_{1}^{'}X_{1}^{'} & \sum X_{1}^{'}X_{2}^{'} & \cdots& \sum X_{1}^{'}X_{p}^{'} \\ \sum X_{2}^{'}X_{1}^{'} & \sum X_{2}^{'}X_{2}^{'} & \cdots& \sum X_{2}^{'}X_{p}^{'} \\ \vdots& \vdots& \ddots& \vdots\\ \sum X_{p}^{'}X_{1}^{'} & \sum X_{p}^{'}X_{2}^{'} & \cdots& \sum X_{p}^{'}X_{p}^{'} \end{matrix}\right]$ (2)

Then, we calculate the eigenvalue and the eigenvector of the covariance matrix by solving the formula (3).

$\left|C-\lambda I\right|=0$ (3)

We compute each component’s contribution. And choose the components top side down with the cumulative contribution larger than 85%. And a correlation analysis between the indicators and the components was conducted. Indicators has the highest correlation coefficient with a component was prior to being considered as the representative indicator. Yet, PCA is not always easy to use as an exclusive method to choose indicators, because in some rare cases the components may have very low correlation with indicators. At this case, we will choose an indicator from the group classified in the correlation analysis by discussion and brainstorming.

**2. Data screening example**

We take the data screening of pressure as an example. Here we omitted the first two steps. We conducted a Pearson correlation analysis for all available data (X1- COD emission of industrial source; X2- TP emission of industrial source; X3- TN emission of industrial source; X4- COD emission of life source; X5- TP emission of life source; X6- TN emission of life source; X7- COD emission of agriculture; X8- TP emission of agriculture; X9- TN emission of agriculture; X10- Total emission of COD; X11- Total emission of TP; X12- Total emission of TN; X13- Non-point source emission of COD; 14- Non-point source emission of TP, X15- Non-point source emission of TN), as the table 1 showed.

Table 1. Pearson correlation analysis of the Pressure indicators

|  | X1 | X2 | X3 | X4 | X5 | X6 | X7 | X8 | X9 | X10 | X11 | X12 | X13 | X14 | X15 |
| --- | --- | --- | --- | --- | --- | --- | --- | --- | --- | --- | --- | --- | --- | --- | --- |
| X1 | 1 | .865** | .814** | -.500 | -.528 | -.582* | -.563 | -.406 | -.151 | -.362 | -.411 | -.507 | -.563 | -.386 | -.151 |
| X2 | .865** | 1 | .930** | -.622* | -.699* | -.738* | -.759** | -.542 | -.377 | -.527 | -.579 | -.678* | -.758** | -.530 | -.377 |
| X3 | .814** | .930** | 1 | -.751** | -.807** | -.842** | -.853** | -.715* | -.587* | -.674* | -.697* | -.796** | -.853** | -.698* | -.587* |
| X4 | -.500 | -.622* | -.751** | 1 | .976** | .939** | .869** | .816** | .888** | .988** | .962** | .951** | .869** | .802** | .888** |
| X5 | -.528 | -.699* | -.807** | .976** | 1 | .984** | .952** | .865** | .896** | .964** | .961** | .993** | .952** | .855** | .896** |
| X6 | -.582* | -.738* | -.842** | .939** | .984** | 1 | .981** | .867** | .857** | .917** | .932** | .994** | .981** | .862** | .857** |
| X7 | -.563 | -.759** | -.853** | .869** | .952** | .981** | 1 | .853** | .781** | .846** | .878** | .971** | 1.000** | .848** | .781** |
| X8 | -.406 | -.542 | -.715* | .816** | .865** | .867** | .853** | 1 | .839** | .814** | .854** | .886** | .853** | .998** | .839** |
| X9 | -.151 | -.377 | -.587* | .888** | .896** | .857** | .781** | .839** | 1 | .933** | .912** | .901** | .781** | .830** | 1.000** |
| X10 | -.362 | -.527 | -.674* | .988** | .964** | .917** | .846** | .814** | .933** | 1 | .969** | .944** | .846** | .802** | .933** |
| X11 | -.411 | -.579 | -.697* | .962** | .961** | .932** | .878** | .854** | .912** | .969** | 1 | .959** | .878** | .851** | .912** |
| X12 | -.507 | -.678* | -.796** | .951** | .993** | .994** | .971** | .886** | .901** | .944** | .959** | 1 | .971** | .880** | .901** |
| X13 | -.563 | -.758** | -.853** | .869** | .952** | .981** | 1.000** | .853** | .781** | .846** | .878** | .971** | 1 | .848** | .781** |
| X14 | -.386 | -.530 | -.698* | .802** | .855** | .862** | .848** | .998** | .830** | .802** | .851** | .880** | .848** | 1 | .830** |
| X15 | -.151 | -.377 | -.587* | .888** | .896** | .857** | .781** | .839** | 1.000** | .933** | .912** | .901** | .781** | .830** | 1 |

** p < 0.01 (single tail); * p < 0.05 (single tail)

Here we can group the indicators into two groups according to there correlations. Group one contains X1-3, and Group two contains X4-15. One indicator has correlation coefficients R > 0.8 with each other indicators in its group.

Then a principal component analysis was conducted and the result was showed in table 2. The result is consistent with the correlation analysis grouping results.

Table 2. Loading matrix of PCA

|  | Component 1 | Component 2 |
| --- | --- | --- |
| X1 | -0.548 | 0.791 |
| X2 | -0.717 | 0.659 |
| X3 | -0.843 | 0.475 |
| X4 | 0.951 | 0.075 |
| X5 | 0.989 | 0.019 |
| X6 | 0.989 | -0.058 |
| X7 | 0.969 | -0.088 |
| X8 | 0.916 | 0.152 |
| X9 | 0.901 | 0.419 |
| X10 | 0.936 | 0.218 |
| X11 | 0.952 | 0.164 |
| X12 | 0.991 | 0.041 |
| X13 | 0.969 | -0.088 |
| X14 | 0.908 | 0.164 |
| X15 | 0.901 | 0.419 |

From table 2, we can choose X12 as the representative indicator for principal component 1, as it has highest correlation coefficient R = 0.991 with component 1. For component 2, we choose X1.
